# Patient knowledge and acceptance of radiation safety measures correlated to actual exposure records: A survey study

**DOI:** 10.6026/973206300221939

**Published:** 2026-04-30

**Authors:** Avinash Nachkunte Lakshmipathy, Zainab Haider Khan, Madhur Gupta, Aisha Mohammed Kutty, Gourav Singh Shekhawat

**Affiliations:** 1 Department of Trauma and Orthopaedics, Aneurin Bevan University Health Board, Wales, United Kingdom; 2 Department of Radiology, Avalon University School of Medicine, Willemstad, Curaçao; 3 Department of Biochemistry, NKPSIMS and Lata Mangeshkar Hospital, Nagpur, India; 4 Department of Oncology and Haematology, Portsmouth University Hospital NHS trust, Wessex, United Kingdom; 5 Department of Community Medicine, Rajasthan University of Health Science and Jai Durga College of Nursing, Rajasthan, India

**Keywords:** Radiation dose optimization, radiological protection practices, diagnostic radiology safety compliance, medical imaging exposure

## Abstract

With the growing prevalence of the use of ionising radiation as a method of medical imaging, it is important to promote patient involvement in the effect of ionising radiation and its relation to medical imaging. Therefore, it is of interest to assess knowledge and acceptance of radiation safety practices amongst 255 adult patients and to compare the results of this survey to patients recorded exposure to radiation. The survey indicated that only 38% of respondents had a high level of understanding of radiation safety practices and only 42% accepted all recommended practices. Patients in the study experienced a mean exposure dose of 4.2 ± 1.8 mSv. Patients with higher levels of knowledge and acceptance of radiation safety practices were associated with less exposure to radiation (p < 0.05) and also predicted decreased exposure to radiation after multivariable adjustment (p < 0.05). Thus, if there is increased structured patient education, this will likely result in better patient compliance with safe use of ionising radiation and will help to optimise radiation exposure.

## Background:

The increasing use of ionising radiation for both medical diagnosis and treatments are associated with many benefits to patients; however, the risks of exposure to ionising radiation as a result of diagnostic procedures can be significant [1]. Studies have shown that many patients do not understand the potential for radiation exposure during diagnostic tests, nor do most patients understand the risks associated with radiation exposure from their diagnostic test [2]. This limited understanding may impact their ability to give informed consent regarding the potential use of ionising radiation during their diagnostic procedure and therefore contribute to not following through with the recommended protective measures such as shielding or asking about the amount of radiation they would be exposed to during their diagnostic procedure [3]. The ALARA principle was established to establish guidelines for using ionising radiation by being justified, optimised and limited to the lowest level possible while still providing necessary diagnostic information [4].

Implementation of patient-focused radiation safety is often not carried out consistently in all locations of healthcare [5]. According to previous studies, both knowledge and attitudes about radiation safety among medical professionals and patients have been shown to have a positive correlation with adherence to radiation safety behaviours [6]. Therefore, it is of interest to determine how much patients know about radiation safety measures and how much they accept them and then correlate these two findings with the actual amount of radiation patients are exposed to during imaging examinations in clinical imaging settings.

## Materials and Methods:

A tertiary care teaching hospital conducted a six-month cross-sectional observational study that evaluated the perceived knowledge of radiation safety, acceptance of behaviour and radiation exposure in adult patients who underwent radiologic or fluoroscopic procedures. Patients aged ≥18 years, who were amenable to being interviewed and provided informed consent, were included in this study. Patients undergoing emergency procedures or had cognitive impairments were excluded from the study. Ethical approval was obtained and all subjects gave written informed consent to participate in the study. Data were collected with a validated structured questionnaire containing demographic data and questions about knowledge of radiation safety and acceptance of protective behaviours. Patients' knowledge about radiation safety was assessed with 15 multiple-choice questions regarding radiation risk and protection. Patients' acceptance of protective behaviours was assessed with 10 behavioural questions regarding the willingness to wear shielding and to request dose information. The radiation exposure dose for each patient was obtained from the hospital's dose management system and patient exposure was matched with patient responses to the questionnaire on a blind, anonymous basis. Descriptive statistics, Pearson correlation, independent t-tests and multiple linear regressions were used to determine predictors of radiation exposure dose. A p-value < 0.05 was considered statistically significant.

## Results:

255 patients were evaluated, an average of 47.6 years of age (±13.2 years) and predominantly female (56). The average knowledge of radiation safety score achieved was (6.4 ±2.1) out of a possible 15, while the average acceptance level for radiological safety was (5.1 ±1.8) out of 10. The average total radiation exposure level recorded was (4.2 ±1.8) mSv. There was a low-to-moderate level of radiation safety knowledge among the participants; only 17.7% achieved a high score on this measure. Patient acceptance of radiological safety was varied, with the majority (82%) of patients willing to wear lead/shielding aprons, while the number of patients requesting dose explanation (32%) or inquiries of radiation risk (46%) was lower. Patients scoring higher on the knowledge of radiation safety scale experienced a significantly lesser average radiation dose than those with a lower knowledge score (3.8 versus 4.6 mSv; p=0.01). Similarly, having a higher level of acceptance of radiological safety was associated with lower average radiation doses than patients having lower levels of acceptance (3.9 versus 4.5 mSv; p=0.02). A positive statistically significant correlation was observed between knowledge and acceptance (r=0.42); conversely, there was an inverse correlation with both radiation dose. Certain demographic characteristics, such as procedure type, knowledge score and acceptance level were identified by multiple regression analysis as significant predictors of radiation dose, with an independently negative relationship between knowledge/acceptance levels and radiation dose. Table 1 (see PDF) depicts the demographic distribution of the study participants (n = 255). The study included a larger proportion of females (56%), with most participants aged 46-60 years (35.7%). Nearly two-thirds (63.5%) of participants indicated they had experience of prior imaging and 40.8% of participants reported education at the graduate or higher level. Table 2 (see PDF) shows the radiation safety knowledge score distribution of participants. Most participants (44.3%) scored in the moderate range, with only 17.7% of participants scoring high. The overall mean score was calculated as 6.4 ± 2.1 out of 15. Table 3 (see PDF) shows participants' attitude towards different radiation safety behaviours and represents a summary of patient engagement of protective behaviours. Most patients accepted fish safety behaviours, like wearing lead aprons (82%) and accept recommendations regarding shielding (74%), but fewer patients performed inquiry to the radiation risk (46%) or requested to have reason provided for their dose (32%). Table 4 (see PDF) represents the average radiation dose among study participants, grouped by the knowledge level and degree of acceptance, with an example of evidence for statically significant negative correlation with knowledge and exposure. Additionally, patients with high knowledge (≥7) and high acceptance (≥5) reported lower average doses (3.8 mSv and 3.9 mSv) respectively than low scoring patients. Table 5 (see PDF) provides the correlation matrix showing relationships between knowledge, acceptance and radiation dose, as well as providing statistical evidence of those relationships. Knowledge and acceptance were positively correlated (r = 0.42) and both knowledge and acceptance presented and inverse correlations (r = -0.26 and -0.31, respectively) with dose suggesting that increased knowledge and acceptance were associated with reduced doses. Table 6 (see PDF) provides multiple regression analysis results for factors associated with reducing radiation dose as well as sensitivity for each variable. It was determined that knowledge (p = 0.02) and acceptance (p = 0.005) higher scores statistically predicted lower doses, while the type of procedure statistically significantly contributed to doses as well (p = 0.001).

## Discussion:

Despite the extensive use of ionising radiation in medical imaging, patients have limited knowledge and acceptance of radiation protection behaviours [7]. Most of the participants showed a moderate level of understanding of the risks associated with radiation exposure and methods of protection, consistent with previous studies that have identified persistent gaps in patient knowledge [1, 2]. There was also a lack of acceptance for the protective behaviours of shielding and dose-related inquiries, indicating that patients are not engaged in safety behaviours related to radiation protection [8]. A major finding of the study is the significant negative correlation between patients’ knowledge and acceptance of radiation safety behaviours and the radiation dose received, indicating that individuals with better awareness and greater willingness to adopt protective measures are exposed to lower levels of ionising radiation [9, 10]. Patients who had higher levels of knowledge about radiation safety and greater acceptance of safety measures received lower doses of radiation and both of these variables independently predicted radiation dose reduction after controlling for demographic factors and procedure type [11]. The data also provides objective evidence that demonstrates the connection between patient education and measurable dose optimisation in the clinical setting [12]. The relationship between knowledge and acceptance indicates that increasing patient knowledge may also lead to increased patient participation in protective behaviours [13]. Through structured pre-procedure counselling, simplified educational materials and patient-centred communication, the level of engagement by patients with respect to radiation safety could be further improved [14]. Involving patients as active participants in the safety culture may also complement the technical optimisation strategies conducted by radiology professionals [15, 16]. This study also helps to clarify the role that procedure type plays in determining the amount of radiation a patient is exposed to, suggesting that both technical factors (e.g., equipment) and behavioural factors (e.g., patient cooperation) work together to determine radiation dose. The limitations of this study (single-centre design and self-reports for measuring acceptance behaviours) point to a need for future multi-centre, interventional studies to assess the long-term benefits of structured educational programs on dose reduction.

## Conclusion:

Patient knowledge and acceptance of radiation safety measures remain suboptimal but are strongly associated with lower radiation exposure. Structured education and active patient engagement should become standard components of institutional radiation protection strategies to optimise dose and strengthen safety culture.
